# CRISPR-Cas: Converting A Bacterial Defence Mechanism into A State-of-the-Art Genetic Manipulation Tool

**DOI:** 10.3390/antibiotics8010018

**Published:** 2019-02-28

**Authors:** Alexandre Loureiro, Gabriela Jorge da Silva

**Affiliations:** 1 Laboratory of Microbiology, Faculty of Pharmacy, University of Coimbra, Health Sciences Campus, Azinhaga de Santa Comba, 3000-548 Coimbra, Portugal; calhaz.al@gmail.com; 2 Center for Neurosciences Cell Biology, University of Coimbra, 3000-548 Coimbra, Portugal

**Keywords:** CRISPR, Cas9, genetic engineering, gene editing

## Abstract

Bacteriophages are pervasive viruses that infect bacteria, relying on their genetic machinery to replicate. In order to protect themselves from this kind of invader, bacteria developed an ingenious adaptive defence system, clustered regularly interspaced short palindromic repeats (CRISPR). Researchers soon realised that a specific type of CRISPR system, CRISPR-Cas9, could be modified into a simple and efficient genetic engineering technology, with several improvements over currently used systems. This discovery set in motion a revolution in genetics, with new and improved CRISPR systems being used in plenty of in vitro and in vivo experiments in recent years. This review illustrates the mechanisms behind CRISPR-Cas systems as a means of bacterial immunity against phage invasion and how these systems were engineered to originate new genetic manipulation tools. Newfound CRISPR-Cas technologies and the up-and-coming applications of these systems on healthcare and other fields of science are also discussed.

## 1. Introduction

Genetic engineering is of great interest for its large array of possible uses in a multitude of scientific domains. This set of technologies enables innovative practices such as the development of drought-resistant plant species [1], the modification of human pluripotent cells [2], or even the generation of genetically modified monkeys [3].

The first attempts to achieve genetically modified organisms (apart from rudimental selective breeding and induced mutagenesis techniques) were unsuccessful up until the 1970s. Transgenesis was effectively used to insert exogenous DNA sequences into *Escherichia coli* plasmids without disrupting the bacteria’s biological functions, resulting in the first genetically modified organism [4].

However, this technique had its limitations. Since it relied on the random insertion of a DNA fragment, there was a risk of mismatch and interference of the exogenous gene with endogenous sequences that were not meant to be altered.

Homologous recombination was the first precise gene-editing technique to be developed [5]. The sequences of the DNA fragment delivered to the cell were homologous to the sequences of a target location in the genome, thus providing a way to reduce non-specific binding. Although this technique could and would be used among the scientific community for research purposes, widespread use was restricted due to its inefficiency.

Over the following decades, the increase in knowledge and information about genetics and advances in DNA sequencing technologies would pave the way for the development of more efficient and precise gene-editing tools, with clustered regularly interspaced short palindromic repeats (CRISPR), as the latest to make the headlines in the scientific community.

## 2. What Is Clustered Regularly Interspaced Short Palindromic Repeats (CRISPR)?

This complex system was first mentioned in 1987 when Japanese scientists were studying the activity of the *iap* gene in *Escherichia coli* [6]. Close to the sequence of the *iap* gene, they noticed an unusual genetic structure composed of alternating repeat and non-repeat DNA sequences, whose biological significance was at the time unclear.

The function of these intriguing systems was only brought to light 20 years later. In a landmark study, experimental evidence established CRISPR as a crucial element of the bacterial defence system against bacteriophage infection [7]. Scientists identified two different CRISPR loci in *Streptococcus thermophilus* strains. Sequencing of the spacer sequences of the CRISPR system revealed that these spacers were homologous to some bacteriophage and plasmid sequences, leading to the hypothesis that CRISPR was a defence mechanism of bacteria against foreign elements. To test this possibility, a phage-sensitive wild-type (WT) *S. thermophilus* strain was challenged with two different virulent bacteriophages. This resulted in the generation of nine different phage-resistant *S. thermophilus* strains, and further analysis of CRISPR loci in these mutant strains discovered that new spacers had been inserted next to those of the WT strain. Additionally, the sequences of these new spacers were similar to sequences within the genome of the phages used in the experiment. This confirmed the hypothesis that, with CRISPR, bacteria submitted to viral stress may integrate new spacers from phage genomic sequences that can lead to a diverse phage resistance phenotype of the bacteria. In the same study, researchers also noticed the proximity of CRISPR sites to a particular set of CRISPR-associated (*cas)* genes that coded Cas proteins, which were also relevant to CRISPR-mediated immunity since silencing of these genes disrupted CRISPR function. 

Bioinformatic databases (CRISPRdb and CRISPI) dedicated to finding CRISPR motifs and Cas proteins on sequenced genomes predict that these systems are prevalent in Archaea (~87%) and can be found on many bacterial genomes and plasmids (~45%) [8,9].

## 3. Structure of CRISPR Loci

Several types of CRISPR exist with varying sequences and reliant on different Cas proteins, although they all share a similar DNA-encoded, RNA-mediated activity. The CRISPR locus, as the full name dictates, is composed of short repeat sequences, usually ranging from 28 to 37 bp (base pairs) [10], separated by spacers each bearing a unique sequence of similar length. Each repeat is arranged in a palindromic fashion, meaning that the repeat’s sequence on one side of the strand is identical to the opposite strand’s sequence when both are read in their respective 5′ to 3′ direction. Spacer sequences feature phage- or plasmid-derived genetic material and constitute the key elements to the specificity of CRISPR’s defene mechanisms [10]. These spacers function as an immunological memory bank, storing sequences from previous encounters with invading organisms. The number of spacers within a CRISPR array can range from as few as one to several hundred, depending on the species [8].

A region rich in adenine and thymine (A and T, respectively), known as the leader sequence, stands upstream to CRISPR loci. These leader sequences have a length of approximately 500 bp and carry promoter elements and signals for CRISPR systems adaptation that are crucial to the transcription of crRNA (CRISPR RNA) and the successful integration of foreign genetic material into CRISPR sequences [11,12].

The CRISPR array and the leader sequence are preceded by CRISPR-associated genes, otherwise known as *cas* genes. Cas proteins (proteins encoded by *cas* genes) pair together with crRNA transcribed from CRISPR loci, forming CRISPR-Cas effector complexes which mediate the silencing and cleavage of alien nucleic acids. Variations in *cas* genes and different arrangements of CRISPR loci originate several types of CRISPR-Cas systems. These were originally broken down into three major types, I, II, and III, each bearing a signature gene specific to the type, *cas3*, *cas9*, and *cas10*, respectively [13]. Recent studies suggest different classifications, with two classes and five [14] or most recently six types of CRISPR-Cas systems [15] having been described. Class 1 systems include types I, III, and IV, which all depend on multiprotein crRNA-complexes to execute their function; class 2 systems encompass types II, V, and VI, whose effector complex is composed of a single multi-domain protein [14,16]. Further subgroups exist within each type, with varied genetic composition and spacer structure.

Cas proteins constitute the backbone of CRISPR systems. The distribution of Cas proteins among different types is highly variable. Some are present in most systems, such as Cas1 and Cas2, which participate in adaptation [17], while others are the signature proteins of specific types or subtypes [18]. Makarova et al. proposed the division of Cas proteins into four modules depending on their roles in CRISPR adaptive immunity: adaptation, for proteins involved in the adaptation step (see below *Adaptation*); expression, with proteins that are required for crRNA maturation and target binding (see below, *CRISPR RNA biogenesis*); interference, for proteins that cleave the target molecules (see below, *Interference*); and ancillary, proteins with regulatory or auxiliary functions in CRISPR systems [14]. The specific functions of the most relevant Cas proteins for each CRISPR-Cas system will be highlighted in the following chapters.

## 4. Steps of CRISPR-Cas Adaptive Immunity

### 4.1. Adaptation

**Figure 1:**
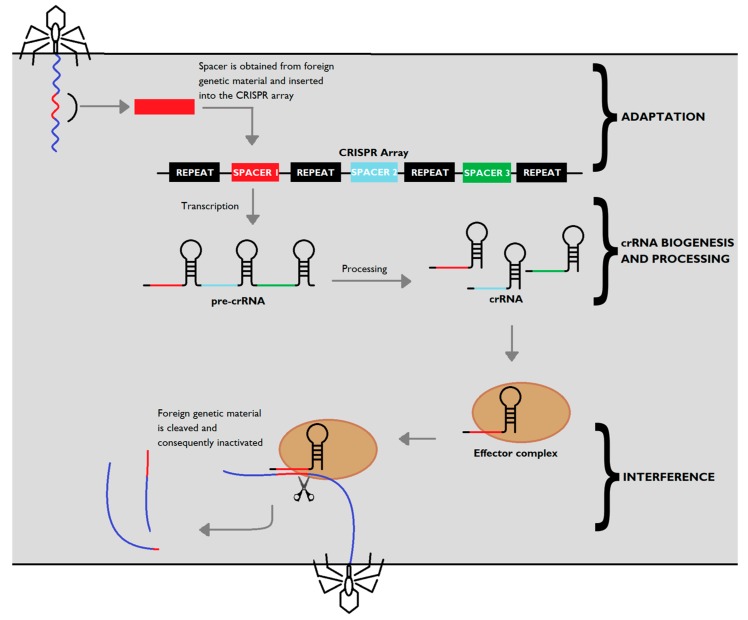
CRISPR-Cas adaptive immunity. Upon injection of genetic material from a virus or a plasmid into the bacteria, part of the invading sequence is cleaved and incorporated into the CRISPR locus, forming a new spacer within the locus. The CRISPR array is transcribed into a precursor to crRNA molecules (pre-crRNA), which is then cleaved into mature crRNA, which form effector complexes with type-specific Cas proteins (brown). When a foreign sequence matches a CRISPR spacer, the matching crRNA binds to the invading strand, activating Cas proteins with nuclease activity which silence the invader.

The first step for CRISPR-Cas mediated defence (Figure 1) is known as adaptation or acquisition. In this phase, genetic material of the invading phage is incorporated into the CRISPR-Cas system, thus providing the organism with a way of recognizing and adjusting its response to further invasions by that phage strain in particular. Cas1 and Cas2 are the key proteins that mediate the adaptation step, and they are ubiquitous in CRISPR-Cas systems, irrespective of the type [17]. Both proteins are required for this step since the expression of Cas1 or Cas2 on their own does not potentiate spacer acquisition [12]. Further experiments showed that mutations on either *cas1* or *cas2* genes in *E. coli* CRISPR sequences render spacer acquisition impossible, while overexpression of both Cas1 and Cas2 improved spacer incorporation [19], confirming the crucial role of both proteins on spacer acquisition. 

In *E. coli,* the most widely studied adaptation model, Cas1 and Cas2 form a symmetric heterohexameric protein complex (Cas1-Cas2) that is required for spacer acquisition, featuring two Cas1 dimers (Cas1a, Cas1a’, Cas1b and Cas1b’) and a single Cas2 dimer [20]. Both proteins have nuclease activity [21,22], although Cas2 nuclease activity is not crucial for spacer acquisition [23]. 

The Cas1-Cas2 complex plays a dual role on the adaptation step, both in the excision of protospacer DNA (segment present in the foreign DNA molecule that precedes the spacer sequence) and its incorporation into the CRISPR sequence [23].

Selection of protospacer sequences seems to be mediated by short motifs located near the target sequence, denominated protospacer adjacent motifs (PAMs). PAMs are short sequences (2–5 nucleotides), specific to each CRISPR-Cas subtype and bacteria, which determine the spacer alignment within the CRISPR array in type 1 and II systems, the better understood models of adaptation [24]. In type I systems, Cas1 binds to the PAM-complementary sequence in its ssDNA form. As for type II systems, Cas9 recognizes the PAM sequence in the double-strand form [25]. Furthermore, PAMs seem to participate in the interference phase and in self/non-self distinction as well [26]. 

Spacer acquisition in the *E. coli* type I-E CRISPR-Cas system begins with the recognition of PAM complementary sequences in ssDNA by Cas1a and Cas1a’ subunits [24]. Tyrosine residues (Tyr22) in Cas1 subunits bracket the foreign genetic material, acting as a ruler that limits the central dsDNA region of the protospacer to a length of 23 nucleotides (nt) [20,27]. The Cas2 dimer of the Cas1-Cas2 complex acts as a stabilizer to the central dsDNA region. Two ssDNA strands at least 7-nt long overhang from each 3′ end of the central duplex region. The last 3 nt (positions 5–7 in the overhangs) correspond to the PAM complementary sequence. These 3 nt are cleaved by nCas1 domains, generating two 3′-OH groups and resulting in a mature protospacer with a length of 33 nt from end to end. 

Integrase activity of the Cas1-Cas2 complex mediates integration of protospacer DNA into the CRISPR array. 3′-OH groups of the protospacer intermediate catalyse 2 sequential nucleophilic attacks at both 5′ ends of the first repeat of the CRISPR array [28]. The result is an expanded CRISPR array with a new spacer in between two incomplete ssDNA repeats, which are afterwards repaired by unknown enzymes. This selection bias for the repeat that is the closest to the leader sequence means that the most recently acquired spacer is the first on the CRISPR array. Therefore, spacers are arranged chronologically within the array, with a few exceptions [29,30]. 

Some type I, II and V systems also rely on Cas4 nuclease activity for the adaptation step [14,18]. The type III-B Cas-system of *Marinomonas mediterranea* is particularly interesting because Cas1 is linked to a reverse transcriptase, meaning that spacers can be obtained from RNA-based invaders and subsequently reverse transcripted into DNA [31]. The need to further comprehend the adaptation machineries for other types of Cas systems persists, although since Cas1 and Cas2 are widely present in nearly all CRISPR systems [17], the function of this complex in most CRISPR systems is thought to be similar to the well understood adaptation mechanisms of type I and type II systems.

### 4.2. CRISPR RNA Biogenesis

The transcription and processing of the CRISPR array and *cas* genes into small crRNAs involves subtype-specific processes and enzymes. In all types of CRISPR-Cas systems, the CRISPR locus is transcribed into a crRNA precursor (pre-crRNA), which is subsequently cleaved and processed by Cas proteins or cellular ribonucleases, yielding smaller units of mature crRNA [32]. This mature crRNA features a single spacer sequence flanked by fragments of the repeat region.

In type I systems, a Cas6 variant (formerly Cse3) is the enzyme that processes the pre-crRNA into mature crRNA fragments. As an example, spacers in type I-E form a stem-loop shape after transcription that is recognised and cleaved by Cas6e. This type I-E specific protein remains attached to the 3′ end of the crRNA after cleavage [33]. Type III systems also require Cas6 for crRNA processing, even though their repeats do not originate in stem-loop structures [34]. 

Type IV CRISPR systems are rare and do not carry the usual proteins CRISPR systems needed for the adaptation and cleavage such as Cas1, Cas2 and Cas4 [14]. Unlike other class 1 systems, type IV lacks Cas6, a protein that types I and III use to process pre-crRNA into mature crRNA. Its multi-subunit effector module is composed of Csf1 (the signature protein of this system), Cas5, and Cas7. Type IV warrants further experimenting to comprehend its mechanisms of adaptation and bacterial immunity.

Type II systems do not carry the gene for Cas6 and instead rely on host RNase III, Cas9 proteins, and small trans-activating RNA molecules (tracrRNA). tracrRNA is complementary to the repeat sequence and contains 3 stem-loop hairpin structures [35,36]. After transcription, tracrRNA binds to pre-crRNA molecules originating dsRNA repeats alternated with ssRNA spacers. Cas9 acts as a molecular anchor which stabilizes the tracrRNA:pre-crRNA interaction for later recognition and cleavage of pre-crRNA by RNase III for complete processing [37].

In type V and type VI, Cas12 and Cas13, respectively, are the proteins that process pre-crRNA into a mature crRNA, without need for tracrRNA molecules [18,38]. However, in subtype VI-A the processing step is not essential, since pre-crRNA molecules can be used as guides for target cleavage [39].

Both type II and III systems require a further trimming step through a ruler-based mechanism for complete crRNA processing. Trimming occurs at the 5′ end in type II systems and at the 3′ end in type III systems [40].

### 4.3. Interference

Upon infection, the mature crRNA molecules direct the subtype-specific interference machinery towards invading nucleic acids to enable the silencing of foreign genetic material.

In type I systems a Cascade system (CRISPR-associated complex for antiviral defence) is formed, composed of a multiprotein backbone with different Cas protein subunits linked to the crRNA molecule [41]. The Cascade complex recognizes the PAM site in the invading molecule and unwinds the DNA, enabling the pairing of crRNA with the homologous invading DNA strand. This pairing induces a triple-stranded R-loop formation, which in turn prompts the recruitment of Cas3, the signature protein of type I systems [42,43]. Cas3 cleaves the ssDNA strand not linked to the Cascade complex. Although this degradation handicaps the invader, it might not lead to the full destruction of the target. Complete degradation might be induced by other cellular nucleases or by Cascade-independent Cas3 nuclease activity, which has been previously documented [44,45,46].

Type III systems are similar to Type I in the sense that they also depend on multiprotein Cascade complexes that encompass crRNA, Csm in subtype III-A and Cmr in III-B, although Cas6 is absent in these complexes [47]. Type III-A and type III-B share in common the signature protein of type III systems, Cas10, and are unique in relation to other interference mechanisms because they target both RNA and DNA substrates [48,49]. Interference by type III systems occurs when the target DNA is being transcripted, since the cascade complex binds to a nascent ssRNA transcript. This binding enables Cas10-mediated cleavage of the complementary DNA duplex and Cas7-guided cleavage of the ssRNA molecule in intervals of 6 nucleotides [47,50]. Recent studies also suggest that Cas10 has a further role in activating non-specific RNase Csm6, by producing cyclic oligoadenylates from ATP molecules. Csm6 is activated by these oligoadenylates, and even though it is not a part of the Cascade effector complex, it has an auxiliary action by degrading foreign transcripts in a non-specific fashion [51,52].

Akin to the biogenesis step, interference in Type II CRISPR-Cas systems depends on both Cas9 and tracrRNA. In interference, Cas9 acts as an endonuclease guided by two RNAs, crRNA and tracrRNA, which pair together due to tracrRNAs complementarity to spacer sequences carried by crRNA, forming a dual RNA complex (tracrRNA:crRNA) [37,53,54]. Binding of this dual RNA structure induces conformational changes on Cas9, leading to its activation [54]. Upon activation, the guide RNA-bound complex screens foreign genetic elements for the correct PAM site, opposite to the target strand. Once identified, the dsDNA is unwinded and crRNA binds to the target ssDNA leading to an R-loop shape and ultimately to a blunt double-strand break by both catalytic sites of Cas9, RuvC and HNH, 3 nt upstream of the PAM site [25,53,54].

Type V CRISPR systems depend on subtype-specific Cas12 proteins, Cas12a (formerly Cpf1), Cas12b and Cas12c for subtypes V-A, V-B, and V-C, respectively [18]. These proteins bear some degree of similarity to Cas9, as noted by phylogenetic analysis and the bilobed structure they share in common [18,55,56]. After PAM site recognition and crRNA binding to target DNA, Cas12a and Cas12b asymmetrically cleave the DNA duplex in both strands, originating staggered breaks with 5- and 7-nt overhangs on Cas12a and Cas12b, accordingly. However, unlike Cas9 or Cas12b, the interference mechanism of Cas12a does not depend on tracrRNA for successful cleaving, and instead relies solely on crRNA [38]. Cas12c still awaits further investigation on its structure and activity.

The recently characterised type VI is defined by the presence of the Cas13 protein (formerly C2c2). This protein contains higher eukaryotes and prokaryotes nucleotide (HEPN)-binding domains, which are ubiquitous in RNases [16,18]. Cas13 is unique relative to other class 2 systems due to its ability to cleave ssRNA molecules homologous to crRNA, which is complemented with non-specific cleaving of other ssRNAs, similar to the Csm6 enzyme of type III systems [57,58]. Cleaving occurs preferably before uridine (U) residues. A species-dependent protospacer flanking site (PFS), analogous to PAMs in DNA targets, is also of relevance for activation of Cas13 proteins [58]. Binding to crRNA induces conformational changes in Cas13 that promote ssRNA pairing. Upon linking to the target, Cas13 RNase activity is prompted by the approximation of the catalytic sites of both HEPN domains [59].

Table 1 highlights the main particularities of each type of CRISPR system.

## 5. CRISPR-Cas Systems as a Gene-Editing Tool

In a landmark paper released in June 2012, Jinek et al. laid the foundation to what would ultimately become a revolution in genome editing and transcriptional control [54]. Jinek and his peers hypothesised that in Type II systems, the dual guide RNA complex tracrRNA:crRNA of Cas9 could be fused into a single chimeric RNA by linking the 3′ end of crRNA to the 5′ end of tracrRNA. Such a technique would allow for programmed DNA cleavage through engineering of the chimeric RNA molecule, later designated as sgRNA or gRNA (guide RNA). This hypothesis was proven with the design five different gRNA molecules to target the green fluorescent protein (GFP) gene, which resulted in precise and efficient cleavage of a plasmid containing the GFP gene by the programmed Cas9 for all five gRNA molecules.

Shortly thereafter, further discoveries would unravel the full potential of CRISPR as a tool for genetic editing. In early 2013, Jiang et al. used the CRISPR-Cas9 system to induce targeted mutations (insertions, deletions, and single-nucleotide substitutions) in the genome of *Streptococcus pneumoniae* and *E. coli* strains [60]. 

Later in the same year, Bikard et al. demonstrated how CRISPR could be used as a new tool to regulate gene expression by either activating or repressing the transcription of bacterial genes [61]. 

As experimentation with CRISPR started to become widespread, scientists moved from bacteria to other kinds of cells. Soon, all sorts of cells and some multicellular organisms would be the object of CRISPR-mediated manipulation, such as human cell cultures, mice, plants, yeasts, and the list goes on [62,63,64,65].

### Repurposing CRISPR for Genetic Engineering

Of both CRISPR-Cas classes, class 2 systems are the most widespread among the scientific community due to the simplicity of their mechanism. Whereas class 1 systems require a convoluted multiprotein Cascade complex, class 2 systems depend only on small RNA molecules, apart from the type’s specific Cas protein [14]. 

**Figure 2:**
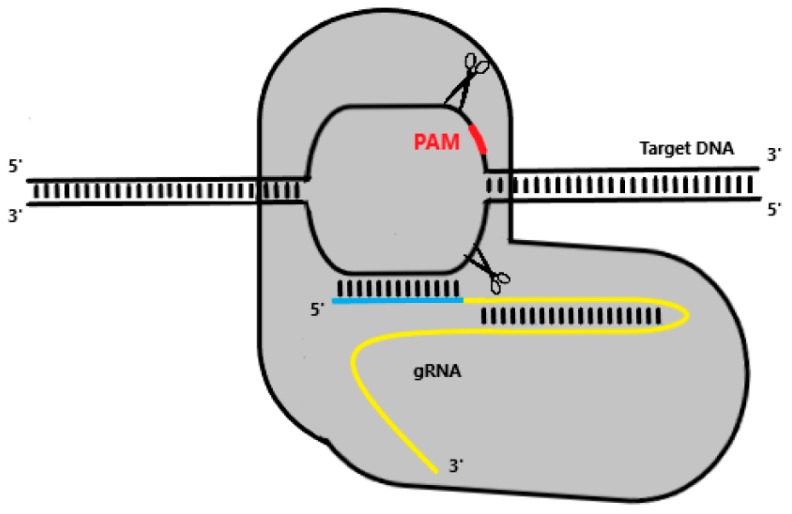
Representation of clustered regularly interspaced short palindromic repeats (CRISPR) Cas9-mediated gene editing. Upon recognising an adequate PAM site (red), the targeting sequence (blue) of gRNA (yellow) begins to anneal to the target DNA in the 3′-5′ direction. If enough homology between strands exists, Cas9 undergoes a conformational change that ultimately results in a blunt DSB.

As previously mentioned (see *Interference*), type II Cas9 systems rely solely on a dual RNA complex of crRNA:tracrRNA, which can be effortlessly engineered into a single chimeric gRNA molecule [54]. gRNA molecules contain both a scaffold sequence that binds to Cas9 and a targeting sequence which directs the system towards the target locus [25]. As Cas9-gRNA screens for a potential target, the first 8–12 PAM-proximal bases of gRNA’s targeting sequence, also known as the seed sequence, will begin pairing with the target DNA in the 3′-5′ direction, provided a PAM site is recognised [66]. While mismatches in the seed sequence terminate pairing and compromise Cas9 cleaving activity, mismatches towards the 5′ PAM-distal end do not always jeopardise Cas9 function [67]. Homology between gRNA and the target sequence results in a double-strand break (DSB) in the DNA, catalysed by both catalytic domains of Cas9, HNH and RuvC (Figure 2). 

DSB repair is mediated either by non-homologous end joining (NHEJ) or by homology-directed repair (HDR). NHEJ is an active and error-prone mechanism where random DNA fragments align with both ends of the DSB and are linked by endogenous repair machinery, provided the bases at both ends share some degree of complementarity [68]. This pathway requires no repair template and constitutes the main route by which Cas9-induced DSBs are repaired. NHEJ can lead to small nucleotide insertions or deletions (indels) in the DSB region, which in turn can originate a vast host of insertions, deletions, or frameshift mutations [69,70]. These mutations derived from Cas9-induced DSBs can be beneficial when trying to attain a knockout in the targeted gene, since indels often result in premature stop codons and consequently render the gene inoperative. However, NHEJ is a highly random and unpredictable process not suitable for the generation of single-base editing or the insertion of specific sequences.

Homology-directed repair arises as a more precise method for DSB repair and incorporation of specific sequences after Cas9 cleavage. Contrarily to NHEJ, HDR requires a DNA template containing the sequence to be delivered to the cell, along with Cas9 and the gRNA [71,72]. For HDR to be successful, both ends of the template must be homologous to the terminal region of the DSB. In order to prevent Cas9 linking and eventual cleavage of the inserted sequence, the PAM sequence should be absent from the repair template. Due to the high efficiency of Cas9 activity and the relatively higher efficiency of NHEJ when compared to HDR, three kinds of entities coexist in this process: wild-type sequences, NHEJ-repaired sequences, and a smaller population of the intended HDR-repaired sequence [73]. Thus, isolation and amplification of the desired sequence are of utmost importance to enhance the in vitro efficiency of HDR.

Apart from mutations and gene editing, CRISPR systems have also been manipulated to increase or reduce gene expression. By introducing two mutations in the RuvC and HNH catalytic domains of Cas9, scientists engineered a ‘dead’ Cas9 (dCas9) that could still bind to DNA but had no cleaving activity [61]. Repression is possible using dCas9 linked to gRNA molecules complementary to a selected gene region. Binding to the targeted gene prevented transcription, seemingly by sterically inhibiting RNA polymerase (RNAP) binding and activity. Moreover, both the initiation and elongation steps of transcription can be prevented through this method, depending on the gene region towards which dCas9 is directed. Another particularly interesting finding was the possibility to modulate the strength of transcription repression by weakening RNA/DNA interactions through the introduction of mismatches in the gRNA/target connection, induced by mutations in the 5′ end of crRNA. Transcription activation was achieved by fusing dCas9 to the omega (ω) subunit of RNAP. dCas9 is directed to the target region, subsequently recruiting and activating the RNAP and culminating in an increase of gene transcription. Novel strategies of Cas9-based transcription modulators rapidly appeared prompted by the fusion of dCas9 or gRNA to different activator or repressor elements, with multiple degrees of modulation and specificity, and enabling single or multiplexed transcriptional control [74,75,76]. Due to its catalytically inactive nature, modifications of gene expression caused by dCas9 are transient, since the genomic DNA is not altered [61,77]. However, persistent modifications can be achieved with dCas9 by fusing it to acetyltransferase, histone/DNA demethylase or methyltransferase, altering histone acetylation/methylation or DNA methylation marks and inducing potentially inheritable epigenetic expression modulation when dividing cells are targeted [78,79,80].

If the purpose is to correct or induce a point mutation requiring only a base substitution, there are simpler methods that do not depend on a DNA template and are more efficient than HDR. By introducing an aspartate-to-alanine (D10A) mutation in the RuvC active domain of Cas9, the resulting mutant, Cas9 nickase, (Cas9n) will nick the target DNA, originating single-stranded breaks rather than DSB [54,81]. Coupling this Cas9n or dCas9, the catalytically “dead” variant of Cas9, with a cytidine deaminase enzyme enables the gRNA-mediated deamination of cytosine (C) bases in the non-target DNA strand into uracil (U), which shares the base-pairing properties of thymine (T) [82]. Through endogenous DNA replication or DNA repair, the U base is repaired to a T base, thereby creating a C→T (or G→A) substitution without inducing a DSB. In the same paper, with enhanced third-generation base editors, Komor et al. achieved >30-fold greater editing efficiency in various human cell lines when compared to HDR-mediated Cas9 editing, with fewer indel formation. Later base editors’ generations built upon this system to increase efficiency and reduce off-target indel formation [83,84,85].

## 6. Advantages of CRISPR Relative to Other Techniques

CRISPR is the latest addition to a set of gene editing tools that keeps evolving and producing new possibilities in the field of genome engineering. Zinc-finger nucleases (ZFN) and transcription activator-like effector nucleases (TALENs) are the other vastly used approaches that complete the lot. 

ZFN and TALENs both derive from the fusion of the DNA cleavage domain of the non-specific *Fok*I restriction endonuclease with DNA-binding elements that direct the enzyme to the desired locus. In ZFNs, the *Fok*I cleaving domain is coupled to an assemblage of zinc finger proteins, each recognizing and binding to a triplet of the nucleotide [86,87]. The design of a ZFN pair targeting opposite strands with an offset of 6 bp results in a DSB in the targeted DNA and can subsequently be repaired by NHEJ or HDR [88]. On the other hand, TALENs were engineered through the fusion of the *Fok*I domain with transcription activator-like (TAL) effectors, proteins found in plant pathogens whose DNA-binding domains (DBD) contain tandem repeats of 33–35 amino acids [89,90]. Each repeat binds to a single nucleotide, and the amino acids residues in the positions 12 and 13 of that repeat determine which nucleotide is bound [91]. By knowing this, it is possible to direct TALENs towards a target locus by programming specific DBDs that can recognize DNA sequences with a length of 15–20 base pairs, originating a DSB when a pair of TALENs is used [92]. 

These two methods have seen use as genetic editing mechanisms, for gene insertion, deletion, and modulation in multiple species and cell lines [93,94,95,96,97]. Nevertheless, both techniques encompass some drawbacks that the use of CRISPR/Cas systems overcomes (Table 2).

For starters, whereas ZFNs and TALENs require custom-made proteins to guide the enzyme towards its target, Cas9 systems depend solely on the engineering of short gRNA molecules, without the need for laborious and costly protein programming and validation and therefore saving time and resources. The need for specific proteins tailored for each gene also makes multiplex gene editing a strenuous task for ZFNs and TALENs, while with Cas9 systems multiple genes can be targeted simply by delivering multiple gRNAs to the cells [63,98]. Additionally, the fact that ZFNs and TALENs work as dimers deters the use of some delivery systems, such as the adeno-associated virus, due to the limited loading capacity of these vectors and the hefty dimensions of ZFN and TALEN systems [99]. The markedly high efficiency of CRISPR/Cas systems coupled with the above-mentioned advantages over other methods justify the CRISPR “epidemic” that the scientific community experienced in 2013 [62,81,100,101].

## 7. Limitations of CRISPR Systems

Despite the qualities of CRISPR systems referred to, some limitations need to be taken into consideration for CRISPR to see use in therapeutic and clinical applications on a larger scale. 

CRISPR-Cas systems require a short PAM sequence next to the 3′ end of the target sequence [24,37]. As an example, the most common Cas9 system, SpCas9, recognises NGG motifs and therefore only sequences adjacent to that motif can be targeted [60]. This feature of CRISPR limits its use when no such PAM exists in the neighbourhood of the locus one wishes to target. However, various conditions attenuate the impact of this obstacle: NGG motifs occur rather frequently, on average every 8 bp in the human genome [81,102]; SpCas9 can also recognise NAG motifs, albeit with lower efficiency [60]; and the fact that Cas9 systems from different bacteria recognise other PAM sites [26], meaning that researchers can pick whatever system better suits their needs. Hu and colleagues recently engineered a SpCas9 variant, xCas9, that recognises additional PAM sites, such as NG, GAA, and GAT, and at the same time displaying significantly lower off-target effects [103].

One of the most significant hurdles that stalls CRISPR adoption is its propensity to generate off-target effects. While TALEN target sites can have a length of ~30 nt, making them unique targets in the genome and lowering the chance of mismatches [104,105], Cas9 is guided by a 20 nt fraction of the gRNA, and it maintains cleaving activity even with 3-5 mismatches at the PAM-distal end of the gRNA molecule [59,81,102]. Defective off-target binding and cleaving can result in collateral mutagenesis induced by the error-prone repair of DSB by NHEJ [106,107]. Harmful consequences can arise from off-target mutations, such as activation of oncogenes or silencing of tumour suppressor genes [108]. New CRISPR variants that minimise off-target effects will be discussed in the next chapter.

Some aspects of HDR of DSBs can also impair CRISPR’s efficiency. For precise gene editing with CRISPR to be a reliable therapeutic alternative, HDR needs to be the main repair mechanism after Cas9-mediated cleavage. Due to the low rate of HDR recombination [109], and because it is only readily available in dividing cells [110,111], this method needs to become more robust and flexible in order to see use in disease therapy. Techniques like synchronisation of cell cycle and use of repair templates of single-stranded oligonucleotide DNA [112], or inhibiting the NHEJ pathway [113] have shown to be useful as means of increasing the efficiency of HDR after Cas9 cleavage. Researchers have also developed a homology-independent targeted integration (HITI) strategy as an alternative to HDR, a technique that allows DNA integration in both dividing and non-dividing cells in vitro and in vivo [114,115].

## 8. Novel and Enhanced CRISPR/Cas Systems

With the aforementioned limitations in mind, researchers have made efforts to improve upon CRISPR systems in order to develop their specificity, efficiency and consistency even further.

### 8.1. Cas12a (Cpf1)

As established previously (See Section 4.3 Interference), type V Cas12a shares some similarities to Cas9 in the sense that it depends only on RNA molecules to originate DSBs, and therefore is classified as a Class 2 CRISPR system [18,38]. However, in contrast to Cas9, it requires only a crRNA molecule to guide it towards its target, in contrast with the crRNA:tracrRNA dual guide of Cas9; and the resulting DSBs are staggered cuts with 5-nt 5′-overhangs as opposed to the blunt cuts generated by Cas9 [116]. Furthermore, while Cas9 enzymes recognise G-rich PAMs, Cas12a preferably links to targets with T-rich PAM sites, this range of recognisable PAMs having increased lately thanks to engineered versions of Cas12a [117]. Additional advantages of Cas12a over Cas9 are the fact that it has lower mismatch tolerance, reducing off-target effects [118]; and Cas12a can process its own crRNA through RNase III activity, thus facilitating multiplex gene editing, as one pre-crRNA template can be delivered to the cell where it is subsequently cleaved by Cas12a into various crRNA molecules targeting different genes [119]. The overhangs left after Cas12a cleaves the target DNA also facilitate HDR, as staggered cuts are preferably repaired through this mechanism rather than NHEJ [120]. Cas12a variants from *Acidaminococcus sp. BV3l6* and *Lachnospiraceae bacterium ND2006*, AsCpf1 and LbCpf1, correspondingly, display similar on-target efficiency to SpCas9 in human cells [121].

### 8.2. Cas13a (C2c2)

The most recent addition to the CRISPR family is particularly unique in comparison to its counterparts. Although type VI Cas13a is also a class 2 CRISPR system, it has the ability to cleave exclusively RNA through the activity of two HEPN domains [58], in contrast with the DNA cleaving ability of Cas9 and Cas12a. It shares with Cas12a the ability to process its own crRNA, which enables the targeting of multiple loci with one pre-crRNA template [122]. The RNA-cleaving properties of Cas13a can be harnessed for post-transcriptional repression, with comparable efficiency to RNA interference (RNAi) methods of RNA silencing [123,124], albeit with more specificity and the ability to cleave nuclear transcripts, that is minimal with RNAi [122]. Due to alternative splicing, the transcription of one DNA sequence results in various splicing isoforms, which means that by targeting DNA with CRISPR systems all mRNA isoforms are affected. By using Cas13a, it is possible to target specifically a single isoform to study its function or interfere with its effect without hampering the activity of the other isoforms [125]. Cas13a can also target pre-mRNA, which can be useful in diseases caused by mis-splicing [126], since the enzyme can act before the defective splicing occurs. However, Cas13a demonstrated indiscriminate RNA cleaving ability [57], which could hinder its usefulness as a therapeutic agent. A recent study found no such effects when the *Leptotrichia wadei* variant, LwaCas13a, was used in mammalian cells [122], implying that this collateral effect might be absent or undetectable in eukaryotic cells.

### 8.3. Cas9n

In situations where gene knockouts are not in order, the NHEJ pathway serves no other purpose than to hinder the repair of DSB by the desired HDR mechanism. As previously stated (see Section 5 Repurposing CRISPR for Genetic Engineering), by introducing a specific mutation in the RuvC domain of Cas9, a Cas9 nickase variant (Cas9n) is created. Cas9n nicks the target DNA, originating single-stranded breaks rather than DSB [54,81]. Single nicked DNA is preferably repaired through base excision repair [127], thus Cas9n can be used to improve the efficiency of the process by reducing the number of indel mutations resulting from unwanted NHEJ repairs. In addition, nickases can be employed to further increase the specificity of Cas9-directed genome editing. Scientists engineered a double nicking scheme featuring a pair of Cas9n targeting opposite strands where neighbouring gRNA targets are offset by a certain number of base pairs [101]. The pairing of Cas9n systems results in DSBs with gRNA-defined overhangs, which can lead to highly specific gene edits when combined with HDR, or originate precise deletions in critical alleles through NHEJ [128,129]. Double nicking dramatically increases specificity, since even if one of Cas9n acts off-target the resulting nick is easily repaired through high-fidelity base excision repair [127], unlike wild-type Cas9, where the blunt off-target DSB can result in undesired mutations when repaired by the NHEJ pathway. However, this method has the drawback of requiring the simultaneous design and delivery of two distinct gRNA molecules.

### 8.4. dCas9

The manipulation of Cas9 systems into tools that modulate gene expression has been previously addressed in this paper in a more detailed fashion (see Section 5 Repurposing CRISPR for Genetic Engineering). When both RuvC and HNH catalytic domains of Cas9 are modified through two silencing mutations, the system loses its DNA cleaving capabilities but retains the ability to bind to targeted sequences [61,77]. Research has demonstrated that this catalytically inactive variant of Cas9 (dCas9) can hinder transcription on its own, presumably by either blocking the pairing between RNA-polymerase and promoter sequences targeted with dCas9, or instead by halting the elongation step if the target sequence is part of an open reading frame region. 

The dCas9 system can be further modified in several ways, such as fusing dCas9 to direct or indirect transcription activators (such as VP64), to increase the expression of a specific DNA sequence; or transcription repressors (such as KRAB), to increase the efficiency of dCas9-mediated transcription inhibition [130,131]. The modification of genetic expression by dCas9 is a transient process, as it does not cause permanent modifications to the genomic DNA. However, specific and long-lasting modifications to genetic expression are possible through the fusion of epigenetic modifiers to dCas9 [78]. In a thorough and enlightening paper, Brocken et al. compiled the most recent advances and strategies for epigenetic modification and transcriptional regulation using dCas9 [132].

### 8.5. eSpCas9, SpCas9-HF1, and HypaCas9

A distinct approach to improve CRISPR targeting specificity relies on the modification of the interactions between the Cas9 system and the bound DNA strands. Slaymaker et al. entertained the possibility that Cas9 cleavage is more efficient when the separation of the target and non-target strands is stable, so undermining this separation in unwanted targets would reduce off-target effects [133]. Upon binding of *Streptococcus pyogenes* Cas9 (SpCas9) to the target site, a stable strand separation is maintained through two kinds of interactions: the binding of gRNA to the target strand, and a positively-charged groove resulting from the unspecific interaction of both HNH and RuvC domains with the negatively-charged non-target strand [36]. Weakening the interactions on the non-target strand by reducing positive charges potentiates the re-hybridization between the target and non-target strand. Off-target effects are therefore reduced since rigorous base pairing between gRNA and the target DNA is required in order to maintain a stable separation of the target and non-target strands. To weaken groove interactions, scientists engineered SpCas9 mutants with a substitution of a single positively-charged amino acid residue, from which resulted two “enhanced specificity” SpCas9 variants (eSpCas9(1.0) and eSpCas9(1.1)), which displayed similar on-target efficiency to WT SpCas9 while having significantly lower levels of off-target cleavage. 

Focusing also on the binding between Cas9 and the target locus, Kleinstiver et al. developed the high-fidelity SpCas9-HF1, a variant that produced undetectable genome-wide off-target cleavage [134]. However, instead of disrupting the non-target strand interactions, Kleinstiver and his colleagues modified four SpCas9 residues that formed hydrogen bonds with the phosphate backbone of the target strand, therefore impairing gRNA binding to DNA targets in the presence of any mismatches. Alanine substitutions in all four residues originated SpCas9-HF1, which along with eSpCas9 also showed comparable on-target activity with WT SpCas9, without impactful off-target effects.

Most recently, Chen et al. utilised single-molecule Förster resonance energy transfer (smFRET) to find out how SpCas9-HF1 and eSpCas9(1.1) differentiate between targets [135]. Throughout their research, scientists have found that SpCas9-HF1 and eSpCas9(1.1) halt in an inactive conformation after they bind to mismatched sequences. Furthermore, they characterised the functions of REC3, a non-catalytic domain of Cas9 that regulates target complementarity and HNH catalytic activity. Using this newfound knowledge, they induced mutations in the REC3 domain, originating a hyper-accurate Cas9 variant (HypaCas9) with the same on-target efficacy as WT Cas9 and similar or improved specificity when compared with SpCas9-HF1 or eSpCas9(1.1).

## 9. Delivering CRISPR Systems into the Cell

One of the most important elements for CRISPR to work is the successful delivery of these systems to the cells that are meant to be altered. Different strategies and techniques have been developed and employed, some with better outcomes for in vitro or in vivo research applications, and others which yield more auspicious results for therapeutic and clinical uses.

Traditional physical methods such as microinjection [136,137] or electroporation [138,139] have been successfully used with CRISPR to engineer embryonic stem cells and zygotes that later originate genetically modified animals. However, microinjection is an intrusive method that can damage the cell and requires the individual injection of each cell, thus constituting a laborious and time-consuming task [140]; and although electroporation is less invasive and allows for the editing of multiple cells at the same time [139], both techniques are only suitable for in vitro use. Hydrodynamic injection is another physical means of gene delivery that has been used to modify liver cells with CRISPR in vivo [141,142]. Even though said techniques are widely used in research labs, these shortcomings combined with low efficiency limit their use in human gene therapy [143,144].

Viral vectors compose a versatile means of delivery with diverse in vitro and in vivo practical applications depending on the chosen vector. For example, adeno-associated viral (AAV) vectors have been used in a dual-cassette system as a way of delivering up to three plasmid-incorporated sgRNAs to the same cell to study gene function in vivo by multiplex gene editing [145] or to create disease models [146]. Due to the different tissue tropism of each AAV serotype, the encapsulated systems can easily be directed towards the tissue of interest by choosing the serotype that better suits the experiment [147]. Genome-wide screening of gene function is another use for viral vectors, through lentiviral gRNA libraries [148,149]. Viral vectors are one of the main means of gene therapy delivery in clinical trials, although their utility and great efficiency might be hindered by several factors, such as immunogenicity, limited insertion capacity (AAV), carcinogenesis (mainly lenti- and retroviruses), and off-target effects (lentiviruses) [99,150,151,152].

Recently developed non-viral vectors have shown promising uses as fitting alternatives to viral and physical methods. As an example for in vitro and ex vivo, cell-penetrating peptides (CPP), conjugated with Cas9 and complexed with gRNA enabled efficient gene silencing in human cell lines and disease models featuring fewer off-target effects when compared to plasmid transfection [153,154]. Another prospect for in vitro and ex vivo CRISPR delivery is through cationic arginine gold nanoparticles (ArgNPs) with engineered Cas9 systems, enhancing the cytosolic delivery of Cas9-gRNA, with an editing efficiency of 30% [155]. As for in vivo, DNA-conjugated gold nanoparticles complexed with endosomal disruptive polymers were used to deliver Cas9, gRNA, and a DNA template to treat Duchenne muscular dystrophy in mice through homology-directed repair, by correcting the mutation that causes this congenital myopathy [156]. Lipid nanoparticles (LNP) are another way to tackle the delivery of CRISPR systems to the cells, with the advantages of being biodegradable and well tolerated. One of such LNP-mediated delivery systems, LNP-INT01, was used with CRISPR to repair the mutated *Ttr* gene that causes transthyretin (TTR)-mediated amyloidosis due to the accumulation of amyloid proteins [157]. A single dose of LNP-INT01 achieved a reduction of more than 97% in serum TTR. 

## 10. Applications of CRISPR-Cas Systems

The versatility and ease of use of the CRISPR methodology, combined with the constant developments to mitigate its flaws, have gathered the attention of researchers from all fields of science who look for ways in which they can use CRISPR to improve and hasten their scientific endeavours. 

### 10.1. Oncology

CRISPR can be used to dissect the diverse genetic and epigenetic factors that are involved in cancer and tumorigenesis. Through HDR- or NHEJ-mediated silencing or knock-in of oncogenes and tumour suppressor genes in vitro, ex vivo, or in vivo, researchers have used CRISPR systems to create cell lines and animal models of certain types of cancer [142,146], as well as to study the impact of specific genes on the progression of the disease [158,159]. Therapeutic uses for CRISPR in cancer are also being researched. As an example, in vivo CRISPR-mediated knockout of *NANOG* and *NANOGP8*, genes involved in prostate cancer, resulted in a decrease of tumorigenic potential in mice [160]. Another example is the knockout of *MDR1* in osteosarcoma cells with Cas9, which reduced cell resistance to chemotherapeutic agents [161]. Clinical trials using CRISPR as a therapeutic agent in cancer are currently underway. In China, researchers from Sichuan University are studying the use of CRISPR-Cas9 for ex vivo engineering of autologous human T-cells, as a treatment for metastatic lung cancer (ClinicalTrials.gov Identifier: NCT02793856). In the United States of America, another clinical trial based on CRISPR-mediated editing of human T-cells is already recruiting, focusing on the treatment of multiple myeloma, sarcoma and melanoma patients (ClinicalTrials.gov Identifier: NCT03399448). 

### 10.2. Genetic Diseases

The correction of diseases arising from genetic aberrations is one of the most obvious use cases for CRISPR methodologies, due to its ability to produce specific changes in the genome. This includes diseases like cystic fibrosis, caused by a mutation in the *CFTR* gene [162], or sickle cell disease, prompted by inheriting two dysfunctional copies of the β-globin (*HBB*) gene, where at least one of those expresses the sickle hemoglobin (HbS) mutation [163]. These inheritable and chronic diseases shorten the life expectancy and quality of life of the carriers [164,165], and can only be attenuated by symptomatic treatments, since the only potential cure available at the moment is stem cell transplant for sickle cell disease [166]. Using CRISPR, researchers were able to correct mutant intestinal stem cells from cystic fibrosis patients and restore their function in vitro [167], providing the first step to advance gene therapy in cystic fibrosis patients with CRISPR. Sickle cell disease has also seen promising breakthroughs, Li and colleagues having successfully corrected the disease-causing mutations in pluripotent stem cells with HDR-mediated Cas9 activity, without noticeable off-target effects [168]. Clinical trial applications for treatment of sickle cell disease using CRISPR/Cas9 systems have already been submitted, and the clinical trials are set to start in 2019 (NCT03745287).

### 10.3. Viral Diseases

CRISPR systems can be employed to combat viral diseases by disrupting the viral replication mechanisms and restoring the infected cell to normality. Cas9 has been used to target conserved regions of the hepatitis B virus (HBV), which are responsible for virus persistence and replication and are not directly targeted by current anti-viral therapies [169]. With Cas9, several research groups targeted this core region of HBV genome, both in vivo and in vitro, successfully supressing the virus with significant and long-lasting reductions in viral load and antigen production, which are related to the severity of the disease [170,171]. Researchers have also eradicated human immunodeficiency virus (HIV-1) from human CD4+ T-cells, by removing parts of its integrated genome using Cas9 [172]. Interestingly, the cured cells were also less susceptible to future infection by HIV-1. CRISPR/Cas13a systems also emerge as an answer to RNA viruses do their capacity to cleave RNA molecules, although due to its novelty ongoing research still focuses on identifying the most potent and specific Cas13 variants [173,174].

### 10.4. Bacterial Infections

The emergence of antimicrobial-resistant bacteria is one of the most concerning aspects for public health specialists nowadays. Misuse and overuse of antibiotics have led to an increasing number of multidrug-resistant strains [175], urging the need for new ways to fight back bacterial infections. CRISPR systems might be an alternative or a tool to use together with conventional antibiotics, either by disabling antibiotic-resistance genes or by developing toxicity in bacteria through the cleavage of crucial domains of their genome, exerting a bactericidal effect [60]. With CRISPR/Cas9, Citorik et al. targeted sequences that enabled antibiotic resistance and virulence in *E. coli* strains [176]. Researchers used Cas9 to target β-lactamase antibiotic resistance genes commonly found in high-copy plasmids in *E. coli.* In a first attempt to deliver this system to the bacteria, the CRISPR machinery was included in a conjugative plasmid. However, this conjugation-based approach resulted in low efficiency, and so researchers turned to bacteriophages as a possible delivery method since they easily inject DNA into particular species of bacteria. The CRISPR system was packaged into phagemid vectors, plasmids that can be loaded into phage capsids [177]. With this method, *E. coli* bearing the antibiotic resistance plasmid sequence were made vulnerable to antibiotic, while causing no unwarranted effects on WT bacteria. In the same paper, using larvae of *Galleria mellonella* (wax moth) as an intestinal infection model, researchers directed Cas9 towards the gene of intimin, a virulence factor of Enterohemorragic *E. coli*. Treatment with Cas9 improved larvae survival and was more effective than treatment with chloramphenicol, an antibiotic to which the *E. coli* strain was resistant.

Later in the same year, a different research group focused on reprogramming CRISPR to target virulence genes in *Staphylococcus aureus* [178]. Using the same phage-based approach to deliver Cas9 systems to bacteria, Bikard et al. targeted the kanamycin resistance gene *aph-3*, a gene carried in the chromosome of strains used in the experiment. This resulted in strong growth inhibition of *S. aureus* due to chromosome cleavage and subsequent cell death. Switching to an in vivo mouse skin colonization model of *S. aureus* infection, treatment with CRISPR also led to a significant reduction of antibiotic-resistant bacteria. 

In both papers, researchers successfully employed the multiplexing capabilities of Cas9 to target multiple chromosomal/plasmid sequences at a time in bacteria [176,178]. This can be useful to target more than one species of bacteria with a single agent, or to affect two different sequences in the same species.

Another aspect highlighted by both research groups is the specificity of CRISPR-based antimicrobials in the treatment of bacterial infections, since it acts selectively on virulent bacteria without affecting the neighboring bacteria. The high degree of specificity is one advantage of this strategy over antibiotics or phages, which kill virulent and innocuous bacteria alike, thereby affecting the microbiota and potentially selecting for resistant bacteria.

Yu et al. suggested a different way in which CRISPR systems can be useful in the fight against bacterial infections [179]. WAP-8294A antibiotics are produced by *Lysobacter* in a very low amount and only under strict conditions. These compounds exhibit potent activity against methicillin resistant *S. aureus*, but the difficulties in obtaining them are an obstacle for researchers. The very low yield is thought to be a self-defense mechanism of *Lysobacter* against the strong activity of these compounds. Building on this, researchers fused dCas9 with a transcription activator to increase the expression of a selected group of genes that had a fundamental role in protecting *Lysobacter* from the action of WAP-8294A compounds. Ultimately, this resulted in a 4- to 9-fold increase in the yield of three WAP-8294A antibiotics.

### 10.5. Crop Industry

Diseases are not the only application for the newfound CRISPR technology, with many industry fields experimenting with CRISPR systems to come up with new methodologies and techniques. One such field is crop science, where researchers consistently breed new varieties of plants to improve agricultural output, confer resistance to certain pathogens, or change specific traits like fruit size. Crop engineering with CRISPR is already in motion, with researchers developing cucumbers with broad virus resistance without affecting plant development [180], improving the yield of maize crops under drought stress [181], or producing seedless tomatoes [182]. The ease of use and precise editing provided by CRISPR systems has the potential to reduce costs and breeding time in crop engineering, improving over current genome editing technologies [183].

## 11. Conclusions

Gene editing techniques have been around for over forty years, yet the limitations affecting their use are still significant in several fields of science. Ethical issues are one of the key concerns among the scientific community, mainly due to the harmful consequences that can result from the genetic manipulation of human and animal germlines. The insufficient precision and efficiency of currently available techniques are also two of the main deterrents against a more widespread use of genetic manipulation.

With this in mind, CRISPR-Cas systems seem to be rapidly changing the landscape of the genetic engineering field. Although initial Cas systems used for genetic engineering were more efficient and simpler than methods such as TALENs and ZFNs, their relatively low specificity and presence of off-target effects meant that they were still not the perfect tool for genetic manipulation.

However, new variants with improved precision and reduced off-target effects while maintaining the original efficiency have been developed and, therefore, the main limitation of these systems has been offset. The applicability of CRISPR-Cas systems is yet to be seen on a larger scale, but the results of upcoming clinical trials using this technology might kick-start new CRISPR “fever”, leading CRISPR systems into mainstream use.

## Figures and Tables

**Table 1:** Characteristics of different types of clustered regularly interspaced short palindromic repeats (CRISPR) systems.

Characteristic	Type I	Type II	Type III	Type IV	Type V	Type VI
Effector complex	Multisubunit (Class 1)	Single unit (Class 2)	Multisubunit (Class 1)	Multisubunit (Class 1)	Single unit (Class 2)	Single unit (Class 2)
Signature Protein	Cas3	Cas9	Cas10	Csf1	Cas12	Cas13
Target molecule	DNA	DNA	RNA/DNA	?	DNA	RNA
Details	Cleaves ssDNA strands	Originates blunt DSB	Binds to nascent RNA molecules	Most unknown CRISPR system	Originates staggered DSB	RNA-guided RNase

**Table 2:** Characteristics of different gene editing tools (Adapted from Chen and Gao 2014).

Characteristic	ZFN	TALEN	CRISPR/Cas9
Binding principle	Protein-DNA	Protein-DNA	RNA-DNA
Ease of design	Moderate	Easy	Very Easy
Assembling	Difficult	Easy	Very Easy
Time for construction	5–7 days	5–7 days	1–3 days
Cost	High	Moderate	Low
Efficiency	Variable	High	High
Off-target effects	High but variable	Low	High
Single-unit or pair	Pair	Pair	Single-unit

## References

[1] Hu H., Xiong L. (2014). Genetic Engineering and Breeding of Drought-Resistant Crops. Annu. Rev. Plant Biol..

[2] Hockemeyer D., Wang H., Kiani S., Lai C.S., Gao Q., Cassady J.P., Cost G.J., Zhang L., Santiago Y., Miller J.C. (2011). Genetic engineering of human pluripotent cells using TALE nucleases. Nat. Biotechnol..

[3] Niu Y., Shen B., Cui Y., Chen Y., Wang J., Wang L., Kang Y., Zhao X., Si W., Li W. (2014). Generation of gene-modified cynomolgus monkey via Cas9/RNA-mediated gene targeting in one-cell embryos. Cell.

[4] Cohen S.N., Chang A.C.Y., Boyer H.W., Helling R.B. (1973). Construction of Biologically Functional Bacterial Plasmids In Vitro. Proc. Natl. Acad. Sci. USA.

[5] Szostak J.W., Orr-Weaver T.L., Rothstein R.J., Stahl F.W. (1983). The double-strand-break repair model for recombination. Cell.

[6] Ishino Y., Shinagawa H., Makino K., Amemura M., Nakata A. (1987). Nucleotide sequence of the iap gene, responsible for alkaline phosphatase isozyme conversion in Escherichia coli, and identification of the gene product. J. Bacteriol..

[7] Barrangou R., Fremaux C., Deveau H., Richards M., Boyaval P., Moineau S., Romero D.A., Horvath P. (2007). CRISPR provides acquired resistance against viruses in prokaryotes. Science (80-.).

[8] Grissa I., Vergnaud G., Pourcel C. (2007). The CRISPRdb database and tools to display CRISPRs and to generate dictionaries of spacers and repeats. BMC Bioinform..

[9] Rousseau C., Gonnet M., Le Romancer M., Nicolas J. (2009). CRISPI: A CRISPR interactive database. Bioinformatics.

[10] Barrangou R., Marraffini L.A. (2014). CRISPR-cas systems: Prokaryotes upgrade to adaptive immunity. Mol. Cell.

[11] Pul Ü., Wurm R., Arslan Z., Geißen R., Hofmann N., Wagner R. (2010). Identification and characterization of E. coli CRISPR-cas promoters and their silencing by H-NS. Mol. Microbiol..

[12] Yosef I., Goren M.G., Qimron U. (2012). Proteins and DNA elements essential for the CRISPR adaptation process in Escherichia coli. Nucleic Acids Res..

[13] Chylinski K., Makarova K.S., Charpentier E., Koonin E.V. (2014). Classification and evolution of type II CRISPR-Cas systems. Nucleic Acids Res..

[14] Makarova K.S., Wolf Y.I., Alkhnbashi O.S., Costa F., Shah S.A., Saunders S.J., Barrangou R., Brouns S.J.J., Charpentier E., Haft D.H. (2015). An updated evolutionary classification of CRISPR-Cas systems. Nat. Rev. Microbiol..

[15] Wright A.V., Nuñez J.K., Doudna J.A. (2016). Biology and Applications of CRISPR Systems: Harnessing Nature’s Toolbox for Genome Engineering. Cell.

[16] Shmakov S., Smargon A., Scott D., Cox D., Pyzocha N., Yan W., Abudayyeh O.O., Gootenberg J.S., Makarova K.S., Wolf Y.I. (2017). Diversity and evolution of class 2 CRISPR-Cas systems. Nat. Rev. Microbiol..

[17] Makarova K.S., Grishin N.V., Shabalina S.A., Wolf Y.I., Koonin E.V. (2006). A putative RNA-interference-based immune system in prokaryotes: Computational analysis of the predicted enzymatic machinery, functional analogies with eukaryotic RNAi, and hypothetical mechanisms of action. Biol. Direct..

[18] Shmakov S., Abudayyeh O.O., Makarova K.S., Wolf Y.I., Gootenberg J.S., Semenova E., Minakhin L., Joung J., Konermann S., Severinov K. (2015). Discovery and Functional Characterization of Diverse Class 2 CRISPR-Cas Systems. Mol. Cell.

[19] Datsenko K.A., Pougach K., Tikhonov A., Wanner B.L., Severinov K., Semenova E. (2012). Molecular memory of prior infections activates the CRISPR/Cas adaptive bacterial immunity system. Nat. Commun..

[20] Nuñez J.K., Harrington L.B., Kranzusch P.J., Engelman A.N., Doudna J.A. (2015). Foreign DNA capture during CRISPR-Cas adaptive immunity. Nature.

[21] Babu M., Beloglazova N., Flick R., Graham C., Skarina T., Nocek B., Gagarinova A., Pogoutse O., Brown G., Binkowski A. (2011). A dual function of the CRISPR-Cas system in bacterial antivirus immunity and DNA repair. Mol. Microbiol..

[22] Wiedenheft B., Zhou K., Jinek M., Coyle S.M., Ma W., Doudna J.A. (2009). Structural Basis for DNase Activity of a Conserved Protein Implicated in CRISPR-Mediated Genome Defense. Structure.

[23] Nuñez J.K., Kranzusch P.J., Noeske J., Wright A.V., Davies C.W., Doudna J.A. (2014). Cas1-Cas2 complex formation mediates spacer acquisition during CRISPR-Cas adaptive immunity. Nat. Struct. Mol. Biol..

[24] Mojica F.J.M., Díez-Villaseñor C., García-Martínez J., Almendros C. (2009). Short motif sequences determine the targets of the prokaryotic CRISPR defence system. Microbiology.

[25] Anders C., Niewoehner O., Duerst A., Jinek M. (2014). Structural basis of PAM-dependent target DNA recognition by the Cas9 endonuclease. Nature.

[26] Shah S.A., Erdmann S., Mojica F.J.M., Garrett R.A. (2013). Protospacer recognition motifs: Mixed identities and functional diversity. RNA Biol..

[27] Wang J., Li J., Zhao H., Sheng G., Wang M., Yin M., Wang Y. (2015). Structural and Mechanistic Basis of PAM-Dependent Spacer Acquisition in CRISPR-Cas Systems. Cell.

[28] Nuñez J.K., Lee A.S.Y., Engelman A., Doudna J.A. (2015). Integrase-mediated spacer acquisition during CRISPR-Cas adaptive immunity. Nature.

[29] Shmakov S., Savitskaya E., Semenova E., Logacheva M.D., Datsenko K.A., Severinov K. (2014). Pervasive generation of oppositely oriented spacers during CRISPR adaptation. Nucleic Acids Res..

[30] Erdmann S., Garrett R.A. (2012). Selective and hyperactive uptake of foreign DNA by adaptive immune systems of an archaeon via two distinct mechanisms. Mol. Microbiol..

[31] Silas S., Mohr G., Sidote D.J., Markham L.M., Sanchez-Amat A., Bhaya D., Lambowitz A.M., Fire A.Z. (2016). Direct CRISPR spacer acquisition from RNA by a natural reverse transcriptase-Cas1 fusion protein. Science (80-.).

[32] Marraffini L.A., Sontheimer E.J. (2010). CRISPR interference: RNA-directed adaptive immunity in bacteria and archaea. Nat. Rev. Genet..

[33] Gesner E.M., Schellenberg M.J., Garside E.L., George M.M., MacMillan A.M. (2011). Recognition and maturation of effector RNAs in a CRISPR interference pathway. Nat. Struct. Mol. Biol..

[34] Niewoehner O., Jinek M., Doudna J.A. (2014). Evolution of CRISPR RNA recognition and processing by Cas6 endonucleases. Nucleic Acids Res..

[35] Chylinski K., Le Rhun A., Charpentier E. (2013). The tracrRNA and Cas9 families of type II CRISPR-Cas immunity systems. RNA Biol..

[36] Nishimasu H., Ran F.A., Hsu P.D., Konermann S., Shehata S., Dohmae N., Ishitani R., Zhang F., Nureki O. (2015). Crystal Structure of Cas9 in Complex with gRNA and target DNA.

[37] Deltcheva E., Chylinski K., Sharma C.M., Gonzales K., Chao Y., Pirzada Z.A., Eckert M.R., Vogel J., Charpentier E. (2011). CRISPR RNA maturation by trans-encoded small RNA and host factor RNase III. Nature.

[38] Fonfara I., Richter H., BratoviÄ M., Le Rhun A., Charpentier E. (2016). The CRISPR-associated DNA-cleaving enzyme Cpf1 also processes precursor CRISPR RNA. Nature.

[39] East-Seletsky A., O’Connell M.R., Burstein D., Knott G.J., Doudna J.A. (2017). RNA Targeting by Functionally Orthogonal Type VI-A CRISPR-Cas Enzymes. Mol. Cell.

[40] Hatoum-Aslan A., Maniv I., Marraffini L.A. (2011). Mature clustered, regularly interspaced, short palindromic repeats RNA (crRNA) length is measured by a ruler mechanism anchored at the precursor processing site. Proc. Natl. Acad. Sci. USA.

[41] Brouns S.J.J., Jore M.M., Lundgren M., Westra E.R., Slijkhuis R.J.H., Snijders A.P.L., Dickman M.J., Makarova K.S., Koonin E.V., van der Oost J. (2008). Small CRISPR RNAs Guide Antiviral Defense in Prokaryotes. Science (80-.).

[42] Xiao Y., Luo M., Hayes R.P., Kim J., Ng S., Ding F., Liao M., Ke A. (2017). Structure Basis for Directional R-loop Formation and Substrate Handover Mechanisms in Type I CRISPR-Cas System. Cell.

[43] Hayes R.P., Xiao Y., Ding F., Van Erp P.B.G., Rajashankar K., Bailey S., Wiedenheft B., Ke A. (2016). Structural basis for promiscuous PAM recognition in type I-E Cascade from *E. coli*. Nature.

[44] Redding S., Sternberg S.H., Marshall M., Gibb B., Bhat P., Guegler C.K., Wiedenheft B., Doudna J.A., Greene E.C. (2015). Surveillance and Processing of Foreign DNA by the Escherichia coli CRISPR-Cas System. Cell.

[45] Mulepati S., Bailey S. (2013). In vitro reconstitution of an Escherichia coli RNA-guided immune system reveals unidirectional, ATP-dependent degradation of DNA Target. J. Biol. Chem..

[46] Sinkunas T., Gasiunas G., Waghmare S.P., Dickman M.J., Barrangou R., Horvath P., Siksnys V. (2013). In vitro reconstitution of Cascade-mediated CRISPR immunity in Streptococcus thermophilus. EMBO J..

[47] Osawa T., Inanaga H., Sato C., Numata T. (2015). Crystal structure of the crispr-cas RNA silencing cmr complex bound to a target analog. Mol. Cell.

[48] Rouillon C., Zhou M., Zhang J., Politis A., Beilsten-Edmands V., Cannone G., Graham S., Robinson C.V., Spagnolo L., White M.F. (2013). Structure of the CRISPR interference complex CSM reveals key similarities with cascade. Mol. Cell.

[49] Kazlauskiene M., Tamulaitis G., Kostiuk G., Venclovas Č., Siksnys V. (2016). Spatiotemporal Control of Type III-A CRISPR-Cas Immunity: Coupling DNA Degradation with the Target RNA Recognition. Mol. Cell.

[50] Samai P., Pyenson N., Jiang W., Goldberg G.W., Hatoum-Aslan A., Marraffini L.A. (2015). Co-transcriptional DNA and RNA cleavage during type III CRISPR-cas immunity. Cell.

[51] Kazlauskiene M., Kostiuk G., Venclovas Č., Tamulaitis G., Siksnys V. (2017). A cyclic oligonucleotide signaling pathway in type III CRISPR-Cas systems. Science (80-.).

[52] Niewoehner O., Garcia-Doval C., Rostøl J.T., Berk C., Schwede F., Bigler L., Hall J., Marraffini L.A., Jinek M. (2017). Type III CRISPR–Cas systems produce cyclic oligoadenylate second messengers. Nature.

[53] Gasiunas G., Barrangou R., Horvath P., Siksnys V. (2012). Cas9-crRNA ribonucleoprotein complex mediates specific DNA cleavage for adaptive immunity in bacteria. Proc. Natl. Acad. Sci. USA.

[54] Jinek M., Chylinski K., Fonfara I., Hauer M., Doudna J.A., Charpentier E. (2012). A Programmable Dual-RNA-Guided DNA Endonuclease in Adaptive Bacterial Immunity. Science (80-.).

[55] Dong D., Ren K., Qiu X., Zheng J., Guo M., Guan X., Liu H., Li N., Zhang B., Yang D. (2016). The crystal structure of Cpf1 in complex with CRISPR RNA. Nature.

[56] Stella S., Alcón P., Montoya G. (2017). Structure of the Cpf1 endonuclease R-loop complex after target DNA cleavage. Nature.

[57] East-Seletsky A., O’Connell M.R., Knight S.C., Burstein D., Cate J.H.D., Tjian R., Doudna J.A. (2016). Two distinct RNase activities of CRISPR-C2c2 enable guide-RNA processing and RNA detection. Nature.

[58] Abudayyeh O.O., Gootenberg J.S., Konermann S., Joung J., Slaymaker I.M., Cox D.B.T., Shmakov S., Makarova K.S., Semenova E., Minakhin L. (2016). C2c2 is a single-component programmable RNA-guided RNA-targeting CRISPR effector. Science (80-.).

[59] Liu L., Li X., Ma J., Li Z., You L., Wang J., Wang M., Zhang X., Wang Y. (2017). The Molecular Architecture for RNA-Guided RNA Cleavage by Cas13a. Cell.

[60] Jiang W., Bikard D., Cox D., Zhang F., Marraffini L.A. (2013). RNA-guided editing of bacterial genomes using CRISPR-Cas systems. Nat. Biotechnol..

[61] Bikard D., Jiang W., Samai P., Hochschild A., Zhang F., Marraffini L.A. (2013). Programmable repression and activation of bacterial gene expression using an engineered CRISPR-Cas system. Nucleic Acids Res..

[62] Ding Q., Regan S., Xia Y., Oostrom L., Cowan C., Musunuru K. (2013). Enhanced efficiency of human pluripotent stem cell genome editing through replacing TALENs with CRISPRs. Cell Stem.

[63] Wang H., Yang H., Shivalila C.S., Dawlaty M.M., Cheng A.W., Zhang F., Jaenisch R. (2013). One-step generation of mice carrying mutations in multiple genes by CRISPR/cas-mediated genome engineering. Cell.

[64] Shan Q., Wang Y., Li J., Zhang Y., Chen K., Liang Z., Zhang K., Liu J., Xi J.J., Qiu J.L. (2013). Targeted genome modification of crop plants using a CRISPR-Cas system. Nat. Biotechnol..

[65] Generoso W.C., Gottardi M., Oreb M., Boles E. (2016). Simplified CRISPR-Cas genome editing for Saccharomyces cerevisiae. J. Microbiol. Methods.

[66] Semenova E., Jore M.M., Datsenko K.A., Semenova A., Westra E.R., Wanner B., van der Oost J., Brouns S.J.J., Severinov K. (2011). Interference by clustered regularly interspaced short palindromic repeat (CRISPR) RNA is governed by a seed sequence. Proc. Natl. Acad. Sci. USA.

[67] Liu X., Homma A., Sayadi J., Yang S., Ohashi J., Takumi T. (2016). Sequence features associated with the cleavage efficiency of CRISPR/Cas9 system. Sci. Rep..

[68] Moore J.K., Haber J.E. (1996). Cell cycle and genetic requirements of two pathways of nonhomologous end-joining repair of double-strand breaks in Saccharomyces cerevisiae. Mol. Cell. Biol..

[69] Bennardo N., Gunn A., Cheng A., Hasty P., Stark J.M. (2009). Limiting the Persistence of a Chromosome Break Diminishes Its Mutagenic Potential. PLoS Genet..

[70] Waters C.A., Strande N.T., Pryor J.M., Strom C.N., Mieczkowski P., Burkhalter M.D., Oh S., Qaqish B.F., Moore D.T., Hendrickson E.A. (2014). The fidelity of the ligation step determines how ends are resolved during nonhomologous end joining. Nat. Commun..

[71] Shrivastav M., De Haro L.P., Nickoloff J.A. (2008). Regulation of DNA double-strand break repair pathway choice. Cell Res..

[72] Pardo B., Gómez-González B., Aguilera A. (2009). DNA double-strand break repair: How to fix a broken relationship. Cell. Mol. Life Sci..

[73] Li K., Wang G., Andersen T., Zhou P., Pu W.T. (2014). Optimization of Genome Engineering Approaches with the CRISPR/Cas9 System. PLoS ONE.

[74] Konermann S., Brigham M.D., Trevino A.E., Joung J., Abudayyeh O.O., Barcena C., Hsu P.D., Habib N., Gootenberg J.S., Nishimasu H. (2015). Genome-scale transcriptional activation by an engineered CRISPR-Cas9 complex. Nature.

[75] Hawkins J.S., Wong S., Peters J.M., Almeida R., Qi L.S. (2015). Targeted transcriptional repression in bacteria using CRISPR interference (CRISPRi). Methods Mol. Biol..

[76] Dominguez A.A., Lim W.A., Qi L.S. (2016). Beyond editing: Repurposing CRISPR–Cas9 for precision genome regulation and interrogation. Nat. Rev. Mol. Cell Biol..

[77] Qi L.S., Larson M.H., Gilbert L.A., Doudna J.A., Weissman J.S., Arkin A.P., Lim W.A. (2013). Repurposing CRISPR as an RNA-Guided Platform for Sequence-Specific Control of Gene Expression. Cell.

[78] Lo A., Qi L. (2017). Genetic and epigenetic control of gene expression by CRISPR–Cas systems. F1000Research.

[79] Amabile A., Migliara A., Capasso P., Biffi M., Cittaro D., Naldini L., Lombardo A. (2016). Inheritable Silencing of Endogenous Genes by Hit-and-Run Targeted Epigenetic Editing. Cell.

[80] Feinberg A.P., Koldobskiy M.A., Göndör A. (2016). Epigenetic modulators, modifiers and mediators in cancer aetiology and progression. Nat. Rev. Genet..

[81] Cong L., Ran F.A., Cox D., Lin S., Barretto R., Habib N., Hsu P.D., Wu X., Jiang W., Marraffini L.A. (2013). Multiplex Genome Engineering Using CRISPR/Cas Systems. Science (80-.).

[82] Komor A.C., Kim Y.B., Packer M.S., Zuris J.A., Liu D.R. (2016). Programmable editing of a target base in genomic DNA without double-stranded DNA cleavage. Nature.

[83] Komor A.C., Zhao K.T., Packer M.S., Gaudelli N.M., Waterbury A.L., Koblan L.W., Kim Y.B., Badran A.H., Liu D.R. (2017). Improved base excision repair inhibition and bacteriophage Mu Gam protein yields C:G-to-T:A base editors with higher efficiency and product purity. Sci. Adv..

[84] Kim Y.B., Komor A.C., Levy J.M., Packer M.S., Zhao K.T., Liu D.R. (2017). Increasing the genome-targeting scope and precision of base editing with engineered Cas9-cytidine deaminase fusions. Nat. Biotechnol..

[85] Rees H.A., Komor A.C., Yeh W.H., Caetano-Lopes J., Warman M., Edge A.S.B., Liu D.R. (2017). Improving the DNA specificity and applicability of base editing through protein engineering and protein delivery. Nat. Commun..

[86] Kim Y.G., Cha J., Chandrasegaran S. (1996). Hybrid restriction enzymes: Zinc finger fusions to Fok I cleavage domain. Proc. Natl. Acad. Sci. USA.

[87] Liu Q., Segal D.J., Ghiara J.B., Barbas C.F. (1997). Design of polydactyl zinc-finger proteins for unique addressing within complex genomes. Proc. Natl. Acad. Sci. USA.

[88] Bibikova M., Golic M., Golic K.G., Carroll D. (2002). Targeted chromosomal cleavage and mutagenesis in Drosophila using zinc-finger nucleases. Genetics.

[89] Christian M., Cermak T., Doyle E.L., Schmidt C., Zhang F., Hummel A., Bogdanove A.J., Voytas D.F. (2010). Targeting DNA double-strand breaks with TAL effector nucleases. Genetics.

[90] Boch J., Scholze H., Schornack S., Landgraf A., Hahn S., Kay S., Lahaye T., Nickstadt A., Bonas U. (2009). Breaking the code of DNA binding specificity of TAL-type III effectors. Science (80-.).

[91] Joung J.K., Sander J.D. (2013). TALENs: A widely applicable technology for targeted genome editing. Nat. Rev. Mol. Cell. Biol..

[92] Miller J.C., Tan S., Qiao G., Barlow K.A., Wang J., Xia D.F., Meng X., Paschon D.E., Leung E., Hinkley S.J. (2011). A TALE nuclease architecture for efficient genome editing. Nat. Biotechnol..

[93] Zhang F., Cong L., Lodato S., Kosuri S., Church G., Arlotta P. (2011). Programmable Sequence-Specific Transcriptional Regulation of Mammalian Genome Using Designer TAL Effectors. Nat. Biotechnol..

[94] Beerli R.R., Dreier B., Barbas C.F. (2000). Positive and negative regulation of endogenous genes by designed transcription factors. Proc. Natl. Acad. Sci. USA.

[95] Segal D.J., Dreier B., Beerli R.R., Barbas C.F. (1999). Toward controlling gene expression at will: Selection and design of zinc finger domains recognizing each of the 5′-GNN-3′ DNA target sequences. Proc. Natl. Acad. Sci. USA.

[96] Orlando S.J., Santiago Y., DeKelver R.C., Freyvert Y., Boydston E.A., Moehle E.A., Choi V.M., Gopalan S.M., Lou J.F., Li J. (2010). Zinc-finger nuclease-driven targeted integration into mammalian genomes using donors with limited chromosomal homology. Nucleic Acids Res..

[97] Cristea S., Freyvert Y., Santiago Y., Holmes M.C., Urnov F.D., Gregory P.D., Cost G.J. (2013). In vivo cleavage of transgene donors promotes nuclease-mediated targeted integration. Biotechnol. Bioeng..

[98] Jao L.-E., Wente S.R., Chen W. (2013). Efficient multiplex biallelic zebrafish genome editing using a CRISPR nuclease system. Proc. Natl. Acad. Sci. USA.

[99] Wu Z., Yang H., Colosi P. (2010). Effect of genome size on AAV vector packaging. Mol. Ther..

[100] Pennisi E. (2013). The CRISPR Craze. Science (80-.).

[101] Mali P., Aach J., Stranges P.B., Esvelt K.M., Moosburner M., Kosuri S., Yang L., Church G.M. (2013). CAS9 transcriptional activators for target specificity screening and paired nickases for cooperative genome engineering. Nat. Biotechnol..

[102] Hsu P.D., Scott D.A., Weinstein J.A., Ran F.A., Konermann S., Agarwala V., Li Y., Fine E.J., Wu X., Shalem O. (2013). Rationally engineered Cas9 nulceases with improved specificity. Nat. Biotechnol..

[103] Hu J.H., Miller S.M., Geurts M.H., Tang W., Chen L., Sun N., Zeina C.M., Gao X., Rees H.A., Lin Z. (2018). Evolved Cas9 variants with broad PAM compatibility and high DNA specificity. Nature.

[104] Juillerat A., Dubois G., Valton J., Thomas S., Stella S., Maréchal A., Langevin S., Benomari N., Bertonat C., Silva G.H. (2014). Comprehensive analysis of the specificity of transcription activator-like effector nucleases. Nucleic Acids Res..

[105] Wefers B., Panda S.K., Ortiz O., Brandl C., Hensler S., Hansen J., Wurst W., Kühn R. (2013). Generation of targeted mouse mutants by embryo microinjection of TALEN mRNA. Nat. Protoc..

[106] Cho S.W., Kim S., Kim Y., Kweon J., Kim H.S., Bae S., Kim J. (2014). Analysis of off-target effects of CRISPR/Cas-derived RNA-guided endonucleases and nickases. Genome Res..

[107] Fu Y., Foden J.A., Khayter C., Maeder M.L., Reyon D., Joung J.K., Sander J.D. (2013). High-frequency off-target mutagenesis induced by CRISPR-Cas nucleases in human cells. Nat. Biotechnol..

[108] Brunet E., Simsek D., Tomishima M., DeKelver R., Choi V.M., Gregory P., Urnov F., Weinstock D.M., Jasin M. (2009). Chromosomal translocations induced at specified loci in human stem cells. Proc. Natl. Acad. Sci. USA.

[109] Wu Y., Liang D., Wang Y., Bai M., Tang W., Bao S., Yan Z., Li D., Li J. (2013). Correction of a genetic disease in mouse via use of CRISPR-Cas9. Cell Stem Cell.

[110] Heyer W.-D., Ehmsen K.T., Liu J. (2010). Regulation of Homologous Recombination in Eukaryotes. Annu. Rev. Genet..

[111] Orthwein A., Noordermeer S.M., Wilson M.D., Landry S., Enchev R.I., Sherker A., Munro M., Pinder J., Salsman J., Dellaire G. (2015). A mechanism for the suppression of homologous recombination in G1 cells. Nature.

[112] Lin S., Staahl B.T., Alla R.K., Doudna J.A. (2014). Enhanced homology-directed human genome engineering by controlled timing of CRISPR/Cas9 delivery. eLife.

[113] Maruyama T., Dougan S.K., Truttmann M.C., Bilate A.M., Ingram J.R., Ploegh H.L. (2015). Increasing the efficiency of precise genome editing with CRISPR-Cas9 by inhibition of nonhomologous end joining. Nat. Biotechnol..

[114] Suzuki K., Tsunekawa Y., Hernandez-Benitez R., Wu J., Zhu J., Kim E.J., Hatanaka F., Yamamoto M., Araoka T., Li Z. (2016). In vivo genome editing via CRISPR/Cas9 mediated homology-independent targeted integration. Nature.

[115] He X., Tan C., Wang F., Wang Y., Zhou R., Cui D., You W., Zhao H., Ren J., Feng B. (2016). Knock-in of large reporter genes in human cells via CRISPR/Cas9-induced homology-dependent and independent DNA repair. Nucleic Acids Res..

[116] Zetsche B., Gootenberg J.S., Abudayyeh O.O., Slaymaker I.M., Makarova K.S., Essletzbichler P., Volz S.E., Joung J., van der Oost J., Regev A. (2015). Cpf1 is a single RNA-guided endonuclease of a class 2 CRISPR-Cas system. Cell.

[117] Gao L., Cox D.B.T., Yan W.X., Manteiga J.C., Schneider M.W., Yamano T., Nishimasu H., Nureki O., Crosetto N., Zhang F. (2017). Engineered Cpf1 variants with altered PAM specificities. Nat. Biotechnol..

[118] Kim D., Kim J., Hur J.K., Been K.W., Yoon S.H., Kim J.S. (2016). Genome-wide analysis reveals specificities of Cpf1 endonucleases in human cells. Nat. Biotechnol..

[119] Zetsche B., Heidenreich M., Mohanraju P., Fedorova I., Kneppers J., DeGennaro E.M., Winblad N., Choudhury S.R., Abudayyeh O.O., Gootenberg J.S. (2016). Multiplex gene editing by CRISPR–Cpf1 using a single crRNA array. Nat. Biotechnol..

[120] Bothmer A., Phadke T., Barrera L.A., Margulies C.M., Lee C.S., Buquicchio F., Moss S., Abdulkerim H.S., Selleck W., Jayaram H. (2017). Characterization of the interplay between DNA repair and CRISPR/Cas9-induced DNA lesions at an endogenous locus. Nat. Commun..

[121] Kleinstiver B.P., Tsai S.Q., Prew M.S., Nguyen N.T., Welch M.M., Lopez J.M., McCaw Z.R., Aryee M.J., Joung J.K. (2016). Genome-wide specificities of CRISPR-Cas Cpf1 nucleases in human cells. Nat. Biotechnol..

[122] Abudayyeh O.O., Gootenberg J.S., Essletzbichler P., Han S., Joung J., Belanto J.J., Verdine V., Cox D.B.T., Kellner M.J., Regev A. (2017). RNA targeting with CRISPR-Cas13. Nature.

[123] Elbashir S.M., Harborth J., Lendeckel W., Yalcin A., Weber K., Tuschl T. (2001). Duplexes of 21 ± nucleotide RNAs mediate RNA interference in cultured mammalian cells. Nature.

[124] Jackson A.L., Bartz S.R., Schelter J., Kobayashi S.V., Burchard J., Mao M., Li B., Cavet G., Linsley P.S. (2003). Off-target gene regulation by RNAi. Nat. Biotechnol..

[125] Mahas A., Neal Stewart C., Mahfouz M.M. (2018). Harnessing CRISPR/Cas systems for programmable transcriptional and post-transcriptional regulation. Biotechnol. Adv..

[126] Scotti M.M., Swanson M.S. (2016). RNA mis-splicing in disease. Nat. Rev. Genet..

[127] Dianov G.L., Hübscher U. (2013). Mammalian base excision repair: The forgotten archangel. Nucleic Acids Res..

[128] Ren X., Yang Z., Mao D., Chang Z., Qiao H.-H., Wang X., Sun J., Hu Q., Cui Y., Liu L.-P. (2014). Performance of the Cas9 Nickase System in *Drosophila melanogaster*. G3.

[129] Shen B., Zhang W., Zhang J., Zhou J., Wang J., Chen L., Wang L., Hodgkins A., Iyer V., Huang X. (2014). Efficient genome modification by CRISPR-Cas9 nickase with minimal off-target effects. Nat. Methods.

[130] Gilbert L.A., Larson M.H., Morsut L., Liu Z., Brar G.A., Torres S.E., Stern-Ginossar N., Brandman O., Whitehead E.H., Doudna J.A. (2013). XCRISPR-mediated modular RNA-guided regulation of transcription in eukaryotes. Cell.

[131] Perez-Pinera P., Kocak D.D., Vockley C.M., Adler A.F., Kabadi A.M., Polstein L.R., Thakore P.I., Glass K.A., Ousterout D.G., Leong K.W. (2013). RNA-guided gene activation by CRISPR-Cas9-based transcription factors. Nat. Methods.

[132] Brocken D.J.W., Tark-Dame M., Dame R.T. (2018). dCas9: A Versatile Tool for Epigenome Editing. Curr. Issues Mol. Biol..

[133] Slaymaker I.M., Gao L., Zetsche B., Scott D.A., Yan W.X., Zhang F. (2016). Rationally engineered Cas9 nucleases with improved specificity. Science (80-.).

[134] Kleinstiver B.P., Pattanayak V., Prew M.S., Tsai S.Q., Nguyen N., Zheng Z., Joung J.K., Unit P., Biology I., Hospital M.G. (2016). High-fidelity CRISPR-Cas9 variants with undetectable genome-wide off-targets. Nature.

[135] Chen J.S., Dagdas Y.S., Kleinstiver B.P., Welch M.M., Sousa A.A., Harrington L.B., Sternberg S.H., Joung J.K., Yildiz A., Doudna J.A. (2017). Enhanced proofreading governs CRISPR-Cas9 targeting accuracy. Nature.

[136] Crispo M., Mulet A.P., Tesson L., Barrera N., Cuadro F., Dos Santos-Neto P.C., Nguyen T.H., Crénéguy A., Brusselle L., Anegón I. (2015). Efficient generation of myostatin knock-out sheep using CRISPR/Cas9 technology and microinjection into zygotes. PLoS ONE.

[137] Horii T., Arai Y., Yamazaki M., Morita S., Kimura M., Itoh M., Abe Y., Hatada I. (2014). Validation of microinjection methods for generating knockout mice by CRISPR/Cas-mediated genome engineering. Sci. Rep..

[138] Véron N., Qu Z., Kipen P.A.S., Hirst C.E., Marcelle C. (2015). CRISPR mediated somatic cell genome engineering in the chicken. Dev. Biol..

[139] Kaneko T., Sakuma T., Yamamoto T., Mashimo T. (2014). Simple knockout by electroporation of engineered endonucleases into intact rat embryos. Sci. Rep..

[140] Zhang Y., Yu L.C. (2008). Single-cell microinjection technology in cell biology. BioEssays.

[141] Yin H., Xue W., Chen S., Bogorad R.L., Benedetti E., Grompe M., Koteliansky V., Sharp P.A., Jacks T., Anderson D.G. (2014). Genome editing with Cas9 in adult mice corrects a disease mutation and phenotype. Nat. Biotechnol..

[142] Xue W., Chen S., Yin H., Tammela T., Papagiannakopoulos T., Joshi N.S., Cai W., Yang G., Bronson R., Crowley D.G. (2014). CRISPR-mediated direct mutation of cancer genes in the mouse liver. Nature.

[143] Valsalakumari J., Baby J., Bijin E., Constantine I., Manjila S., Pramod K. (2013). Novel gene delivery systems. Int. J. Pharm. Investig..

[144] Kamimura K., Suda T., Zhang G., Liu D. (2011). Advances in gene delivery systems. Pharmaceut. Med..

[145] Swiech L., Heidenreich M., Banerjee A., Habib N., Li Y., Trombetta J., Sur M., Zhang F. (2015). In vivo interrogation of gene function in the mammalian brain using CRISPR-Cas9. Nat. Biotechnol..

[146] Platt R.J., Chen S., Zhou Y., Yim M.J., Swiech L., Kempton H.R., Dahlman J.E., Parnas O., Eisenhaure T.M., Jovanovic M. (2014). CRISPR-Cas9 Knockin Mice for Genome Editing and Cancer Modeling. Cell.

[147] Zincarelli C., Soltys S., Rengo G., Rabinowitz J.E. (2008). Analysis of AAV serotypes 1-9 mediated gene expression and tropism in mice after systemic injection. Mol. Ther..

[148] Shalem O., Sanjana N.E., Hartenian E., Shi X., Scott D.A., Heckl D., Ebert B.L., Root D.E., Doench J.G. (2014). Genome-scale CRISPR-Cas9 knockout screening in human cells. Science (80-.).

[149] Koike-Yusa H., Li Y., Tan E.P., Velasco-Herrera M.D.C., Yusa K. (2014). Genome-wide recessive genetic screening in mammalian cells with a lentiviral CRISPR-guide RNA library. Nat. Biotechnol..

[150] Somia N., Verma I.M. (2000). Gene therapy: Trials and tribulations. Nat. Rev. Genet..

[151] Biasco L., Baricordi C., Aiuti A. (2012). Retroviral integrations in gene therapy trials. Mol. Ther..

[152] Holkers M., Maggio I., Henriques S.F.D., Janssen J.M., Cathomen T., Gonçalves M.A.F.V. (2014). Adenoviral vector DNA for accurate genome editing with engineered nucleases. Nat. Methods.

[153] Ramakrishna S., Kwaku Dad A.B., Beloor J., Gopalappa R., Lee S.K., Kim H. (2014). Gene disruption by cell-penetrating peptide-mediated delivery of Cas9 protein and guide RNA. Genome Res..

[154] Suresh B., Ramakrishna S., Kim H. (2017). Eukaryotic Transcriptional and Post-Transcriptional Gene Expression Regulation. Methods Mol. Biol..

[155] Mout R., Ray M., Yesilbag Tonga G., Lee Y.W., Tay T., Sasaki K., Rotello V.M. (2017). Direct Cytosolic Delivery of CRISPR/Cas9-Ribonucleoprotein for Efficient Gene Editing. ACS Nano.

[156] Lee K., Conboy M., Park H.M., Jiang F., Kim H.J., Dewitt M.A., Mackley V.A., Chang K., Rao A., Skinner C. (2017). Nanoparticle delivery of Cas9 ribonucleoprotein and donor DNA in vivo induces homology-directed DNA repair. Nat. Biomed. Eng..

[157] Finn J.D., Smith A.R., Patel M.C., Shaw L., Youniss M.R., van Heteren J., Dirstine T., Ciullo C., Lescarbeau R., Seitzer J. (2018). A Single Administration of CRISPR/Cas9 Lipid Nanoparticles Achieves Robust and Persistent In Vivo Genome Editing. Cell Rep..

[158] Matano M., Date S., Shimokawa M., Takano A., Fujii M., Ohta Y., Watanabe T., Kanai T., Sato T. (2015). Modeling colorectal cancer using CRISPR-Cas9-mediated engineering of human intestinal organoids. Nat. Med..

[159] Chen S., Sanjana N.E., Zheng K., Shalem O., Shi X., Scott D.A., Song J., Pan J.Q., Weissleder R., Zhang F. (2016). Genome-wide CRISPR screen in a mouse model of tumor growth and metastasis. Cell.

[160] Kawamura N., Nimura K., Nagano H., Yamaguchi S., Nonomura N., Kaneda Y. (2015). CRISPR/Cas9-mediated gene knockout of NANOG and NANOGP8 decreases the malignant potential of prostate cancer cells. Oncotarget.

[161] Liu T., Shen J.K., Li Z., Choy E., Hornicek F.J., Duan Z. (2016). Development and potential applications of CRISPR-Cas9 genome editing technology in sarcoma. Cancer Lett..

[162] Kerem B., Rommens J.M., Buchanan J.A., Markiewicz D., Cox T.K., Chakravarti A., Buchwald M., Tsui L.C. (1989). Identification of the cystic fibrosis gene: Genetic analysis. Science.

[163] Steinberg M.H., Sebastiani P. (2012). Genetic modifiersof sickle cell disease. Am. J. Hematol..

[164] Ratjen F., Döring G. (2003). Cystic fibrosis. Lancet.

[165] Lanzkron S., Carroll C.P., Haywood C. (2013). Mortality rates and age at death from sickle cell disease: U.S., 1979–2005. Public Health Rep..

[166] Özdoğu H., Boğa C. (2015). Erişkin orak hücre hastalığında hematopoietik kök hücre nakli: Problemler ve çözüm önerileri. Turk. J. Hematol..

[167] Schwank G., Koo B.K., Sasselli V., Dekkers J.F., Heo I., Demircan T., Sasaki N., Boymans S., Cuppen E., Van Der Ent C.K. (2013). Functional repair of CFTR by CRISPR/Cas9 in intestinal stem cell organoids of cystic fibrosis patients. Cell Stem Cell.

[168] Li C., Ding L., Sun C.W., Wu L.C., Zhou D., Pawlik K.M., Khodadadi-Jamayran A., Westin E., Goldman F.D., Townes T.M. (2016). Novel HDAd/EBV Reprogramming Vector and Highly Efficient Ad/CRISPR-Cas Sickle Cell Disease Gene Correction. Sci. Rep..

[169] Zeisel M.B., Lucifora J., Mason W.S., Sureau C., Beck J., Levrero M., Kann M., Knolle P.A., Benkirane M., Durantel D. (2015). Towards an HBV cure: State-of-the-art and unresolved questions-report of the ANRS workshop on HBV cure. Gut.

[170] Lin S.R., Yang H.C., Kuo Y.T., Liu C.J., Yang T.Y., Sung K.C., Lin Y.Y., Wang H.Y., Wang C.C., Shen Y.C. (2014). The CRISPR/Cas9 system facilitates clearance of the intrahepatic HBV templates in vivo. Mol. Ther. Nucleic Acids.

[171] Kennedy E.M., Bassit L.C., Mueller H., Kornepati A.V.R., Bogerd H.P., Nie T., Chatterjee P., Javanbakht H., Schinazi R.F., Cullen B.R. (2015). Suppression of hepatitis B virus DNA accumulation in chronically infected cells using a bacterial CRISPR/Cas RNA-guided DNA endonuclease. Virology.

[172] Kaminski R., Chen Y., Fischer T., Tedaldi E., Napoli A., Zhang Y., Karn J., Hu W., Khalili K. (2016). Elimination of HIV-1 Genomes from Human T-lymphoid Cells by CRISPR/Cas9 Gene Editing. Sci. Rep..

[173] Cox D.B.T., Gootenberg J.S., Abudayyeh O.O., Franklin B., Kellner M.J., Joung J., Zhang F. (2017). RNA editing with CRISPR-Cas13 David. Science (80-.).

[174] Mahas A., Mahfouz M. (2018). Engineering virus resistance via CRISPR–Cas systems. Curr. Opin. Virol..

[175] Thabit A.K., Crandon J.L., Nicolau D.P. (2015). Antimicrobial resistance: Impact on clinical and economic outcomes and the need for new antimicrobials. Expert Opin. Pharmacother..

[176] Citorik R.J., Mimee M., Lu T.K. (2014). Sequence-specific antimicrobials using efficiently delivered RNA-guided nucleases. Nat. Biotechnol..

[177] Melnikov A.A., Tchernov A.P., Fodor I., Bayev A.A. (1984). Lambda phagemids and their transducing properties. Gene.

[178] Bikard D., Euler C.W., Jiang W., Nussenzweig P.M., Goldberg G.W., Duportet X., Fischetti V.A., Marraffini L.A. (2014). Exploiting CRISPR-Cas nucleases to produce sequence-specific antimicrobials. Nat. Biotechnol..

[179] Yu L., Su W., Fey P.D., Liu F., Du L. (2018). Yield Improvement of the Anti-MRSA Antibiotics WAP-8294A by CRISPR/dCas9 Combined with Refactoring Self-Protection Genes in Lysobacter enzymogenes OH11. ACS Synth. Biol..

[180] Chandrasekaran J., Brumin M., Wolf D., Leibman D., Klap C., Pearlsman M., Sherman A., Arazi T., Gal-On A. (2016). Development of broad virus resistance in non-transgenic cucumber using CRISPR/Cas9 technology. Mol. Plant Pathol..

[181] Shi J., Gao H., Wang H., Lafitte H.R., Archibald R.L., Yang M., Hakimi S.M., Mo H., Habben J.E. (2017). ARGOS8 variants generated by CRISPR-Cas9 improve maize grain yield under field drought stress conditions. Plant Biotechnol. J..

[182] Ueta R., Abe C., Watanabe T., Sugano S.S., Ishihara R., Ezura H., Osakabe Y., Osakabe K. (2017). Rapid breeding of parthenocarpic tomato plants using CRISPR/Cas9. Sci. Rep..

[183] Jaganathan D., Ramasamy K., Sellamuthu G., Jayabalan S., Venkataraman G. (2018). CRISPR for Crop Improvement: An Update Review. Front. Plant Sci..

